# Missing heritability, polygenic scores, and gene–environment correlation

**DOI:** 10.1111/jcpp.12128

**Published:** 2013-09-05

**Authors:** Robert Plomin

**Affiliations:** MRC Social, Genetic & Developmental Psychiatry Centre, Institute of Psychiatry, King’s College LondonLondon, UK

## Abstract

This special issue amply fulfils its aim of moving the study of gene × environment (GE) interplay forward constructively and creatively, exploiting contributions from diverse disciplines. Rather than discussing the many interesting findings and methods in this special issue, I will comment on two cross-cutting issues – one about genes and the other about the environment – that came to mind as I read these articles.

This special issue amply fulfils its aim of moving the study of gene × environment (GE) interplay forward constructively and creatively, exploiting contributions from diverse disciplines. Rather than discussing the many interesting findings and methods in this special issue, I will comment on two cross-cutting issues – one about genes and the other about the environment – that came to mind as I read these articles.

## Missing heritability and the need for polygenic scores

Despite the breath-taking advances in molecular genetics and genomics, DNA research has not yet delivered the genes that developmentalists are eager to incorporate in their research (Plomin, 2013). What we have learned from the whirlwind of genome-wide association (GWA) studies during the past few years is that the biggest effect sizes for associations between genes and traits – both for common disorders and quantitative dimensions – are much smaller than anyone expected. For example, in a GWA meta-analysis of IQ for nearly 18,000 children, the largest effect size accounts for 0.2% of the variance (Benyamin et al., 2013). If the largest effects are so small, the smallest effects will be infinitesimal, which means that they will be difficult to detect in GWA studies and even harder to replicate. Although it is difficult to reach the power needed to detect such small effects, the studies are powered to detect a modest effect size, which means that this strong conclusion can be drawn for complex traits: There are no effect sizes greater than 1%.

Finding such miniscule effect sizes in GWA studies conflicts with hundreds of candidate gene and GE interaction studies that find significant effects on behaviour with modest sample sizes powered only to detect large effects. The concern is that underpowered candidate gene reports of significant associations and GE interactions are false positives that will not replicate, both for main effects and interactions. This is the reason why some journals now require that candidate gene papers include an independent replication. (See *Winham & Biernacka*^1^).

Because so many genes of such small effect are responsible for the heritability of behavioural traits, their practical use in developmental research will require aggregation in polygenic scores, in which genotypes at many loci are summed (Plomin & Simpson, 2013). When polygenic scores are available, they will transform research on GE interplay because their greater effect sizes will make it possible to conduct adequately powered research that can detect reliable results with reasonable sample sizes. For example, a polygenic score constructed from 32 single nucleotide polymorphisms (SNPs) shown to be associated with body mass index (BMI) has been used in several studies. One developmental study, for instance, found that this BMI polygenic score, which had been derived from studies of adults, is associated with weight gain from birth to age 3 but not with birth weight (Belsky et al., 2012). In the first developmental study using polygenic scores, a polygenic score for 5 SNPs associated with IQ at age 7 showed significant GE interaction for parental discipline, education and occupation in which the association between the polygenic score and IQ at age 7 was stronger in low-risk environments (Harlaar et al., 2005), similar to the quantitative genetic analysis reported in this special issue that genetic influences are stronger in low-risk environments (*Burt, Klahr, Neale, & Klump*). However, the early GWA results on which this polygenic score was derived have only been partially replicated.

Polygenic scores have been created for many medical disorders and some psychiatric disorders, but the problem is that effect sizes for individual SNPs are so small that even these aggregate scores explain only a small amount of variance. For example, the polygenic score for BMI explains less than 2% of the variance of BMI. The tiny effect sizes have led to a problem that plagues not just behavioural research but all genomic research in the life sciences: the *missing heritability problem*, which refers to the wide gap between the heritability of a trait and the variance accounted for in total by known gene associations (Plomin & Simpson, 2013). Much has been written about the possible causes of the gap – such as the need to study rarer DNA variants (current DNA arrays used in GWA research only genotype common SNPs) – and ways to close the gap – such as analysing whole genes and gene networks rather than individual DNA variants (see *Winham & Biernacka*).

There are two reasons for optimism that bigger and better polygenic scores will emerge. First, a new quantitative genetic technique that estimates heritability using DNA alone suggests that about half of the heritability of many complex traits can be detected using the common SNPs that are currently genotyped on commercially available DNA arrays given sufficiently large samples (Plomin, Haworth, Meaburn, Price, & Davis, 2013). Second, the field is waiting for the next major development, whole-genome sequencing, which genotypes all 3 billion base pairs of DNA for each individual and in this way identifies DNA sequence variation of every kind throughout the genome (Plomin & Simpson, 2013).

A practical problem for developmentalists is that a polygenic score requires genotyping many DNA variants, not just a few candidate genes. Rather than genotyping a few candidate genes one by one, it would seem to make sense to use a DNA array that genotypes a million SNPs and allows imputation of nearly any common DNA variant in the genome. Genotyping with a DNA array costs no more than genotyping a few candidate genes. However, the problem with DNA arrays is that they are limited to common SNPs, whereas hope for closing the missing heritability gap lies in part with rarer variants and variants other than SNPs. For this reason, the ultimate strategy is whole-genome sequencing because no more genotyping ever needs to be performed once the entire DNA sequence is known. Although whole-genome sequencing currently costs a few thousand dollars, the costs are declining rapidly and are expected to fall below $1000 (Plomin & Simpson, 2013).

The most exciting long-term possibility is that it may cost nothing to obtain whole-genome sequencing for huge samples of infants! In an excellent book on the potential of the genomics revolution for personalised medicine, Francis Collins, former director of the Human Genome Project and currently director of the U.S. National Institutes of Health, has predicted: “I am almost certain that complete genome sequencing will become part of newborn screening in the next few years. It is likely that within a few decades people will look back on our current circumstance with a sense of disbelief that we screened for so few conditions” (Collins, 2010, p.50). If this prediction is correct and access to children’s DNA sequence was possible, it will be a game-changer for developmentalists. It would no longer be necessary to collect DNA, to genotype it, or to sequence it – we can just use it. Developmental researchers could use any combination of DNA variants to create polygenic scores to use as a predictor of children’s genetic propensities, which will make it possible to trace how those genetic dispositions develop longitudinally, how they overlap with other traits, and how they interact and correlate with the environment.

## GE correlation as well as GE interaction

The amazing advances in DNA research in the last few years have been led by new technology, especially DNA arrays that can genotype a million SNPs and now whole-genome sequencing (Plomin, 2013). I wish there were comparable breakthroughs for research on the environmental side of gene-environment interplay. There are interesting parallels between genes and environments. For example, polygenic effects (each trait is affected by many genes) are mirrored in poly-environmental effects, and polygenic scores are mirrored in poly-environmental scores seen in this special issue in attempts to construct environmental risk composites (*Burt* et al.*; Hudson* et al.). There are also parallels with genetic pleiotropy (each gene affects many traits) – many environmental risk factors are also likely to have pervasive effects in development. However, studying the environment is much more difficult than studying genes because the environment is not based on a simple molecule like DNA with its triplet code. One far-reaching possibility is that environmental research could capitalise on the advances in whole-genome technology to identify biomarkers of environmental influence using genome-wide gene expression (transcriptomics; *Wolock* et al.) and DNA methylation (epigenomics; *Hudson* et al.; *Lewis* et al.; Plomin & Simpson, 2013).

Although this special issue makes it clear that there is much to do in terms of understanding GE interaction, I suggest that GE correlation will in the end be more enlightening about the developmental interplay between genes and environment. GE interaction denotes genetically driven sensitivity to environments. In other words, the effect of the environment on a phenotype depends on genotype. GE correlation is a very different way of thinking about the interplay between genes and environment. GE correlation literally denotes a correlation between genotypes and environments; it has been described as genetic control of exposure to the environment. In the developmental interplay between environments and outcomes, GE interaction moderates the association whereas GE correlation mediates the association. GE interaction and GE correlation assume different models of the environment. The GE interaction model assumes an environment ‘out there’ that is imposed on the organism, although the effects of the environment on developmental outcomes depend on the genotype of the organism. The essence of active GE correlation is choice: individuals select, modify and create experiences that are correlated with their genetic propensities.

Animal model research focuses on GE interaction because of the ability in the laboratory to impose exactly the same environment on each individual animal. GE correlation is not studied in the laboratory because the need to give animals environmental choices defeats the purpose of experimental control provided in the laboratory. The model of imposed environments is also the reason why the star of GE interaction research is pharmacogenetics: An administered drug is the prototype of an imposed environment. However, even in the case of pharmacogenetics, the key to individual differences in the use and abuse of drugs is not the pharmacological properties of the drugs but individuals’ choices about using drugs. GE interaction studies in human development investigate naturally occurring environmental factors such as parenting that cannot be controlled as in a laboratory experiment. For this reason, GE correlation comes into these GE interaction analyses but only as something that needs to be controlled to study GE interaction (e.g., *Chen, Li, & McGue*).

The importance of GE correlation became clear in the 1980s when it was found that most measures ostensibly assessing psychologically relevant aspects of the environment such as parenting and life events in fact show substantial genetic influence. For example, a review of 55 independent genetic studies using environmental measures found an average heritability of 27% across 35 different environmental measures (Kendler & Baker, 2007). Although environments per se cannot show genetic influence, measures of the environment can show genetic influence to the extent that they are correlated genetically with characteristics of individuals such as behavioural traits. Recent research has gone beyond demonstrating genetic influence on environmental measure to investigating genetic mediation between environmental measures and developmental outcomes. Quantitative genetic studies have been used to identify true environmental effects while controlling for genetic effects, as well as providing examples of passive and evocative types of GE correlation (*Harold* et al.; Knafo & Jaffee, 2013). DNA studies are also attempting to identify genes associated with environmental measures and that mediate associations between environment and outcome.

I suggest that *active* GE correlation is key to understanding the developmental GE interplay by which genotypes use the environment – from cells to society – to develop into phenotypes. Existing research provides only a glimpse of this world of the genetics of experience because extant environmental measures implicitly assume a passive model of the environment imposed from the outside, while extant behavioural measures assess characteristics within the individual. GE correlation research has made an important contribution by showing that genetic factors contribute to these environmental measures and mediate associations between these environmental and behavioural measures. But what we need are strategies to move beyond passive models and measures of imposed environments to investigate how individuals actively construct their experiences as a function of their genetic propensities.

## Footnotes

1 Articles in this issue (with italicised author names on first mention) can be found at http://onlinelibrary.wiley.com/doi.10.1.1.1.1/jccp.2013.54.issue-10/issuetoc.
